# Transforming physical fitness and exercise behaviors in adolescent health using a life log sharing model

**DOI:** 10.3389/fpubh.2025.1562151

**Published:** 2025-04-04

**Authors:** Shanshan Wang, Jingwu Liu

**Affiliations:** Department of Physical Education, Changzhi University, Changzhi, Shanxi, China

**Keywords:** life log, behavior classification, deep learning, physical activity, public health, multimodal data analysis

## Abstract

**Introduction:**

This study investigates the potential of a deep learning-based Life Log Sharing Model (LLSM) to enhance adolescent physical fitness and exercise behaviors through personalized public health interventions.

**Methods:**

We developed a hybrid Temporal–Spatial Convolutional Neural Network-Bidirectional Long Short-Term Memory (TS-CNN-BiLSTM) model. This model integrates temporal, textual, and visual features from multimodal life log data (exercise type, duration, intensity) to classify and predict physical activity behaviors. Two datasets, Geo-Life Log (with location data) and Time-Life Log (without location data), were constructed to evaluate the impact of spatial information on classification performance. The model utilizes CNNs for local feature extraction and BiLSTM networks to capture temporal dynamics, maintaining user privacy.

**Results:**

The TS-CNN-BiLSTM model achieved an average classification accuracy of 99.6% across eight physical activity types, outperforming state-of-the-art methods by 1.9–4.4%. Temporal features were identified as crucial for detecting recurring behavioral trends and periodic exercise patterns.

**Discussion:**

These findings demonstrate the efficacy of integrating multimodal life log data with deep learning for accurate physical activity classification. The high accuracy of the TS-CNN-BiLSTM model supports its potential for developing personalized health promotion strategies, including tailored interventions, behavioral incentives, and social support mechanisms, to enhance adolescent engagement in physical activities and advance public health education and personalized health management.

## 1 Introduction

With the rising obesity rate and the popularity of sedentary lifestyles among adolescents around the world, adolescents’ physical health exercise behaviors have become a focus of attention in the field of public health (1). Adolescent physical inactivity has emerged as a critical global public health challenge, driven by rising obesity rates and sedentary lifestyles 11. Recent studies highlight stark disparities: for example, low-income regions report 30% lower physical activity engagement compared to high-income areas, while socioeconomic factors (e.g., access to facilities) further exacerbate this gap. Physical activity is not only crucial for adolescents’ physical health, but also has a profound impact on their mental health and social adjustment (2). However, how to effectively motivate and sustain physical activity participation among adolescents is a challenge for educators, parents, and public health professionals (3).

The rapid development of information technology has provided new solutions to the problem of physical activity among adolescents, and in particular, the LLSM has shown significant potential in health promotion (4). The LLSM is an innovative intervention model based on a digital platform that integrates personal exercise data recording, information sharing, social interaction, and behavioral feedback (5). The LLSM addresses the challenge of motivating and sustaining physical activity among adolescents through several innovative mechanisms that traditional methods often lack. Firstly, the LLSM leverages digital platforms to integrate personal exercise data recording, information sharing, social interaction, and behavioral feedback. This holistic approach allows for real-time monitoring and dynamic data sharing, which can significantly enhance adolescents’ intrinsic motivation and sense of competition. Unlike traditional methods that may rely on periodic assessments or static feedback, the LLSM provides continuous and interactive engagement. Additionally, the use of mobile terminals and wearable devices ensures that data collection is both accurate and convenient, making it easier for adolescents to track their activities and receive immediate feedback. This real-time interaction and social support network can foster a more sustained interest in physical activity compared to traditional interventions that may not offer such personalized and dynamic engagement. Real-time data sharing enables adolescents to monitor exercise progress, compare achievements with peers, and foster intrinsic motivation through social competition (6).

Artificial intelligence, particularly neural networks, plays a crucial role in enhancing the effectiveness of the LLSM for adolescent health promotion (7). Neural networks provide a powerful tool for extracting deep features and recognizing patterns from complex life log data (8). By analyzing large amounts of data collected through the LLSM, neural networks can identify individual behavioral patterns, predict health outcomes, and offer personalized health advice to adolescents (9). This capability is particularly significant because it allows for tailored interventions that can adapt to the unique needs and preferences of each individual. For example, the fusion of CNN (Convolutional Neural Networks) and BiLSTM (Bidirectional Long Short-Term Memory) models can capture both spatial and temporal features from life log data, enabling more accurate classification and prediction of physical activity behaviors. This level of personalization and precision is difficult to achieve with traditional methods, making AI a vital component in optimizing the LLSM for adolescent health promotion.

However, existing interventions and models often fall short in several key areas: (1) Limited Personalization: Traditional methods typically rely on generalized recommendations and static feedback, which fail to account for individual differences in physical activity patterns, preferences, and health conditions. This lack of personalization reduces the effectiveness of interventions in motivating sustained engagement. (2) Inadequate Temporal and Contextual Analysis: Many existing models focus on spatial or static features (e.g., location or activity type) but overlook the importance of temporal dynamics (e.g., time of day, duration, and frequency of activities). This limits their ability to capture the nuanced behavioral patterns that are critical for effective health interventions. (3) Privacy Concerns: Current models often require extensive personal data, including sensitive location information, raising privacy concerns, especially among adolescent users. This can hinder user adoption and compliance with health interventions. (4) Lack of Multimodal Data Integration: Most existing approaches rely on single-modal data (e.g., text or images), which limits their ability to comprehensively analyze physical activity behaviors. The integration of multimodal data (e.g., temporal, textual, and visual features) is essential for accurate classification and prediction but remains underexplored in current solutions.

To address these limitations, this propose a hybrid TS-CNN-BiLSTM model that integrates temporal, textual, and visual features from multimodal life log data. This study aims to develop and evaluate a deep learning-based LLSM to improve physical fitness behaviors in adolescents by leveraging multimodal data and advanced neural network techniques. The proposed model offers several key innovations: (1) Enhanced Personalization: By leveraging deep learning techniques, the model can analyze individual behavioral patterns and provide tailored exercise recommendations based on personal health status and preferences. This personalized approach enhances user engagement and adherence to physical activity programs. (2) Temporal and Contextual Insights: The model incorporates temporal features (e.g., time of day, duration) to capture dynamic trends in adolescent exercise behavior. This allows for more accurate classification and prediction of physical activities, enabling targeted interventions that align with users’ daily routines. (3) Privacy-Preserving Design: The model is designed to operate effectively even without sensitive location information, as demonstrated by the Time-Life Log dataset. This ensures user privacy while maintaining high classification accuracy, addressing a critical barrier to adoption. (4) Multimodal Data Integration: By combining CNN for local feature extraction and BiLSTM for temporal dynamics, the model effectively integrates multimodal data to achieve robust classification and prediction. This holistic approach overcomes the limitations of single-modal models and provides a more comprehensive understanding of physical activity behaviors.

Structure overview of the paper: Section 1 analyzes the current state of adolescent physical activity and limitations of existing models, proposing the TS-CNN-BiLSTM hybrid model; Section 2 summarizes log analysis techniques and health applications; Section 3 details dual-dataset construction (Geo/Time-Life Log), privacy-preserving preprocessing, and the CNN-BiLSTM spatio-temporal feature fusion architecture; Section 4 (Result analysis and discussion) validates the model’s accuracy and generalization capabilities; Section 5 proposes personalized intervention strategies; Section 6 highlights innovations and future directions (e.g., interpretability).

## 2 State of the art

Logs are used to record events that occur at each moment in chronological order (10). In computers, logs can be a record of events such as access operations (time, type of operation, and user) to the computer (11). Logging is very important and when a computer malfunctions, it can be analyzed and trouble shooted based on the events recorded in the log files (12).

The concept of Artificial Intelligence for IT Operations (AIOps) was introduced by applying artificial intelligence to the field of operation and maintenance (13). It leverages large-scale data such as logs and monitoring information, utilizing machine learning and other algorithms to automatically detect and respond to system issues in real time, thereby enhancing the level of automated operation and maintenance. AIOps significantly improves efficiency and effectiveness compared to traditional IT operations in three key aspects: (1) Proactive fault detection: Traditional methods rely on manual monitoring and reactive troubleshooting, which often delay issue resolution. In contrast, AIOps analyzes log patterns through machine learning to identify anomalies (e.g., abnormal program behaviors) and diagnose root causes with fine-grained precision, reducing downtime by up to 40%. (2) Predictive insights: AIOps employs predictive analytics to forecast potential system failures (e.g., server overloads) based on historical data trends, enabling preemptive mitigation before issues escalate. (3) Automated Response: By integrating automated workflows (e.g., self-healing scripts), AIOps resolves common issues without human intervention, accelerating resolution times from hours to minutes. These advancements address limitations of conventional approaches, such as fragmented data analysis and delayed responses, making AIOps a transformative tool for modern IT ecosystems.

Log data compared to other data can support more fine-grained root cause diagnosis of faults (14), tracking and capturing abnormal programs (15) and other characteristics, log analysis technology has become a current research hotspot. From the daily use of operating systems, website applications to various software applications on cell phones, a large amount of log data is generated every day. Logs record detailed operational information, with logging code updated at a frequency roughly one time faster than other code segments. On average, there is one line of logging code in about 30 lines of code, which shows that the number of logs generated is huge and needs to be updated from time to time. Program developers pay more attention to the update and maintenance of logging code, logging can help developers to maintain the system more easily (16). Making full use of log data and automated analysis can maximize the value of logs and be applied to many fields, such as root cause analysis, system anomaly detection, behavioral analysis, etc. based on logs.

Life log is personal data created by an individual’s life experiences and behaviors in daily life, which includes location, behaviors, audio and pictures (17). This data reveals what happened to a person when and where, and by further analyzing the data, we are also able to analyze the user’s daily behavior (18). There are a wide range of applications for life log data, including sports data analytics, digital healthcare, smart home, etc. (19). Life log is a phenomenon where people can digitally record different details of their daily lives for various purposes.

Life logs have received attention from academia and industry as data reflecting life experiences that are passively collected and processed through multimedia sensors. A team of researchers from Lanzhou University published an important study in the journal European Review of Aging and Physical Activity that examines the impact of a healthy lifestyle on delaying aging and reducing the risk of all-cause mortality (20). The study, based on data from the UK Biobank, reveals how lifestyle habits such as an anti-inflammatory diet, moderate physical activity and good sleep play an important role in the aging process (21). The findings emphasize the importance of a good lifestyle for improving quality of life and prolonging lifespan. Beccaluva et al. (22) utilized a large number of audio, phonological, and lexical features to characterize events in everyday audio streams. They used voice activity detection and speaker dialing system for high-level semantic segmentation of audio files and proposed a new method to analyze and classify daily activities in personal recordings. Chaturvedi et al. (23) used a collected dataset containing life log and music information. They combined the life log information with audio and music metadata and proposed a model based on 2D Thayer emotion detection. Experiments proved that the user information based classification method can effectively recognize music emotions. Echtioui et al. (24) extracted specific features from life logs and used these features for artificial neural network based classification. Ben-Dor et al. (25) proposed a new direction for self-awareness using life journals, revealing an important relationship between daily activity logging and physical and psychological self-awareness. By analyzing, predicting, and intervening in an individual’s physical and mental state, we can more accurately understand the individual’s condition and obtain effective health advice, and even detect physical and mental abnormalities at an early stage. Hoang-Xuan et al. (26) proposed a method for classifying life logs based on textual topics and geographic location. The method splices the extracted text topic features and geolocation features with the text dynamics and inputs them into the Text CNN model for classification. The experimental results show that the classification method that incorporates geographic features and topic features improves the level of understanding of the text content, thus improving the accuracy of the classification of life logs.

## 3 Methodology

### 3.1 Description of the dataset

#### 3.1.1 Liu-Life Log

In this paper, we utilize the Liu-Life Log dataset for the methodology and application of log classification prediction. The Liu-Life Log project was started in 2011 and covers 26 different categories of daily behaviors, such as work, rest, study, and physical activities. It reflects the adolescents’ patterns of physical activity at different time periods. For an illustrative overview of this dataset’s content, refer to the entries presented in Table 1.

**Table 1:** Example of Liu-Life Log.

Cluster	Date	Time	Describe	Address	Behavior
1	2024/4/10	14:00	Played basketball with friends at the school gym.	School Gym, Beijing, China	School sports
2	2024/4/12	16:30	Participated in a soccer match against Team B.	Local Soccer Field, Shanghai	Competitive sports
3	2024/4/15	18:45	Completed a 3 km run in the community park.	Community Park, Guangzhou	Physical exercise
4	2024/4/17	10:00	Attended a morning yoga session at the park.	City Park, Shenzhen	Fitness activity
5	2024/4/19	15:45	Joined a cycling club for a weekend ride.	Starting Point, Hangzhou	Recreational cycling

#### 3.1.2 Data preprocessing

Raw life log data underwent preprocessing to standardize input dimensions and enhance model accuracy. Noisy data (e.g., empty entries from system errors or incomplete submissions) were removed. Temporal inconsistencies in user-generated logs were corrected to ensure continuity. In order to accomplish the classification and prediction tasks, all the noisy data from the Li-Life Log needs to be cleaned. The missing empty data in the dataset are identified and processed, and a deletion strategy is adopted to remove specific formats and meaningless data, remove duplicates and erroneous data, and ensure the consistency and accuracy of the data.

When constructing the LLSM for youth physical activity behavior, we pay special attention to user privacy protection, especially considering the sensitivity of youth users to personal location information. Therefore, we extracted and constructed two complementary data subsets from the Liu-Life Log dataset: the Geo-Life Log and the Time-Life Log. Geo-Life Log contains location information (e.g., city, address) along with other features such as image labels, video labels, and date. It was created by extracting records from the Liu-Life Log dataset that included geographic data. The location information was used to analyze the spatial patterns of adolescents’ physical activities. Time-Life Log dataset excludes location information to ensure user privacy. It includes features such as image labels, video labels, and date. The Time-Life Log was generated by removing all geographic data (e.g., city, address) from the Liu-Life Log dataset, focusing solely on temporal and activity-related features. These two data subsets, although originating from the same dataset, each contain different features, and the specific details are shown in Table 2.

**Table 2:** Details of the dataset.

Dataset	Geo-Life Log	Time-Life Log
Feature composition	Position, city, describe, image labels, video labels, date	Image labels, describe, video labels, date
Training set/entries	9,850	
Test set/entries	2,500	
Validation set/entries	2,500	
Category	26	

The analysis revealed correlations between adolescents’ daily physical activities and specific time periods. For example, by analyzing the large number of records in the life log dataset, we found that most of the logs uploaded by adolescents in the afternoon after school were related to physical activities, and the corresponding behavior labels were “in the school playground” or “in the community playground.” Therefore, the introduction of temporal features in the model construction process is crucial to capture the time-related activity patterns. This helps to improve the model’s ability to predict youth physical activity.

To ensure the fairness and comparability of the experimental design, we extracted 9,850 records from each of the two data subsets as the training set, while 2,500 records were assigned to each of the validation and test sets. Both sub-datasets were preprocessed to remove noisy data, such as empty entries, duplicates, and erroneous records, ensuring data consistency and accuracy. The image and video data were converted into textual tags using the Recognize Everything Model (RAM) (27), and video data were preprocessed to select the most representative frames for feature extraction. The model not only performs well in image tagging, but also shows strong performance in zero-sample generalization ability. RAM is able to automatically recognize over 6,400 commonly used tags, covering a wider range of categories than OpenImagesV6. With this technique, we convert image content in our life log into textual tags. The working code for recognize-anything can be found on GitHub. For video data, we first perform preprocessing, including video resizing, cropping, and frame rate control. Then the video images are segmented by combining Bayesian decision making method and inter-frame differencing, from which the most representative frames are selected as the feature representation of the video.

The study ensures the fairness and comparability of the experimental design by carefully constructing the training, validation, and test sets for both the Geo-Life Log and Time-Life Log datasets. Specifically, 9,850 records were extracted from each dataset to form the training set, while 2,500 records were assigned to each of the validation and test sets. This consistent allocation of data ensures that both datasets have an equal number of records for each phase of the experiment, which helps in maintaining fairness. Additionally, both datasets were preprocessed to remove noisy data, such as empty entries, duplicates, and erroneous records, ensuring data consistency and accuracy. This preprocessing step is crucial for maintaining comparability, as it ensures that the datasets are of high quality and free from biases that could affect the model’s performance. By following these rigorous preprocessing and allocation steps, the study ensures that the experimental design is fair and comparable across both datasets.

### 3.2 TS-CNN-BiLSTM model

The proposed TS-CNN-BiLSTM model innovatively integrates CNN’s local feature extraction capability with BiLSTM’s temporal modeling strengths, overcoming the limitations of individual models (e.g., CNN or LSTM alone) in spatio-temporal feature fusion. Compared to existing hybrid models, its uniqueness lies in achieving high classification accuracy under privacy-preserving conditions (without location data) through collaborative analysis of multimodal data (text + time + visual), significantly outperforming traditional methods.

The model constructed in this paper aims to provide an accurate activity classification of physical activity behaviors involved in adolescents’ multimodal life logs. The model synthesizes and analyzes information from different data sources to identify and differentiate the various physical activities that adolescents engage in during their daily lives. Firstly, the text is disambiguated and tokenized using the Tokenizer disambiguator, which converts the text into sequences with each word corresponding to a token. The tokenized text is converted into a word embedding representation using an Embedding layer. The processed word vectors are then passed through the CNN layer and local features in the text are extracted using Convolutional and Pooling layers. This helps the model to capture important information and features in the text. Then the extracted local features are passed through BiLSTM layer which is used to model the contextual connections in the text. BiLSTM is able to efficiently process time-series data and capture long distance dependencies. Through the combination of CNN and BiLSTM, the model is able to capture the global relationships of the whole sentence, which leads to a better understanding of the semantic information of the text. Finally, the obtained text representation is passed into the fully connected layer for activity classification. The classification results are mapped to (0,1) probability intervals by SoftMax function to output the corresponding activity categories.

#### 3.2.1 Word embedding

Word embedding is the process of converting a word or sub word into a vector representation. In natural language processing, a word or sub word is usually represented as a sparse vector of high dimensions, where each dimension corresponds to a feature of the word or sub word. For example, in a word list containing 100 words, each word can be represented as a sparse vector of size 100. Only one of the dimensions is 1 and the rest of the dimensions are 0. This high-dimensional sparse representation not only wastes storage space but also makes it difficult to compute the similarity between words. Therefore, word embedding techniques are used to map words or sub words into a low dimensional dense vector space so that the similarity between words can be represented and computed more efficiently.

Building on these advantages, the TS-CNN-BiLSTM model leverages word embeddings to enhance physical activity classification through four key mechanisms: (1) Semantic representation: Word embeddings capture semantic relationships between words (e.g., “running” and “jogging” are mapped to similar vectors), enabling the model to recognize contextual nuances in physical activity descriptions. (2) Dimensionality reduction: By converting sparse one-hot vectors into dense representations (e.g., 300 dimensions), computational efficiency is improved while preserving semantic information. (3) Contextual understanding: The BiLSTM layer leverages sequential dependencies in embedded vectors to model temporal patterns (e.g., distinguishing “morning jog” from “evening cycling”). (4) Generalization: Embeddings allow the model to infer meanings of unseen but semantically related words (e.g., “sprinting” in test data is mapped close to “running” in training data), enhancing robustness. These capabilities collectively improve the model’s ability to classify physical activity behaviors with higher accuracy and interpretability.

A life log is usually composed of a series of words or sub-words. The task of a Tokenizer is to separate these words or sub-words from the text and convert them into a numerical representation that can be processed by a computer. The tokenization algorithm converts text into a sequence of words or sub-words. Based on Tokenizer the text can be converted into a separate list of tokens, which in turn can be converted into input vectors into a computer understandable form of input. Segmenters and word embedding techniques are often used jointly in classification tasks to convert text into a numeric representation that can be processed by a computer. The text is divided into sequences of words using a lexer; then each word is represented as a vector using word embedding techniques; and finally these vectors are fed into the neural network for classification.

#### 3.2.2 TS-CNN-BiLSTM model framework

**Figure 1:**
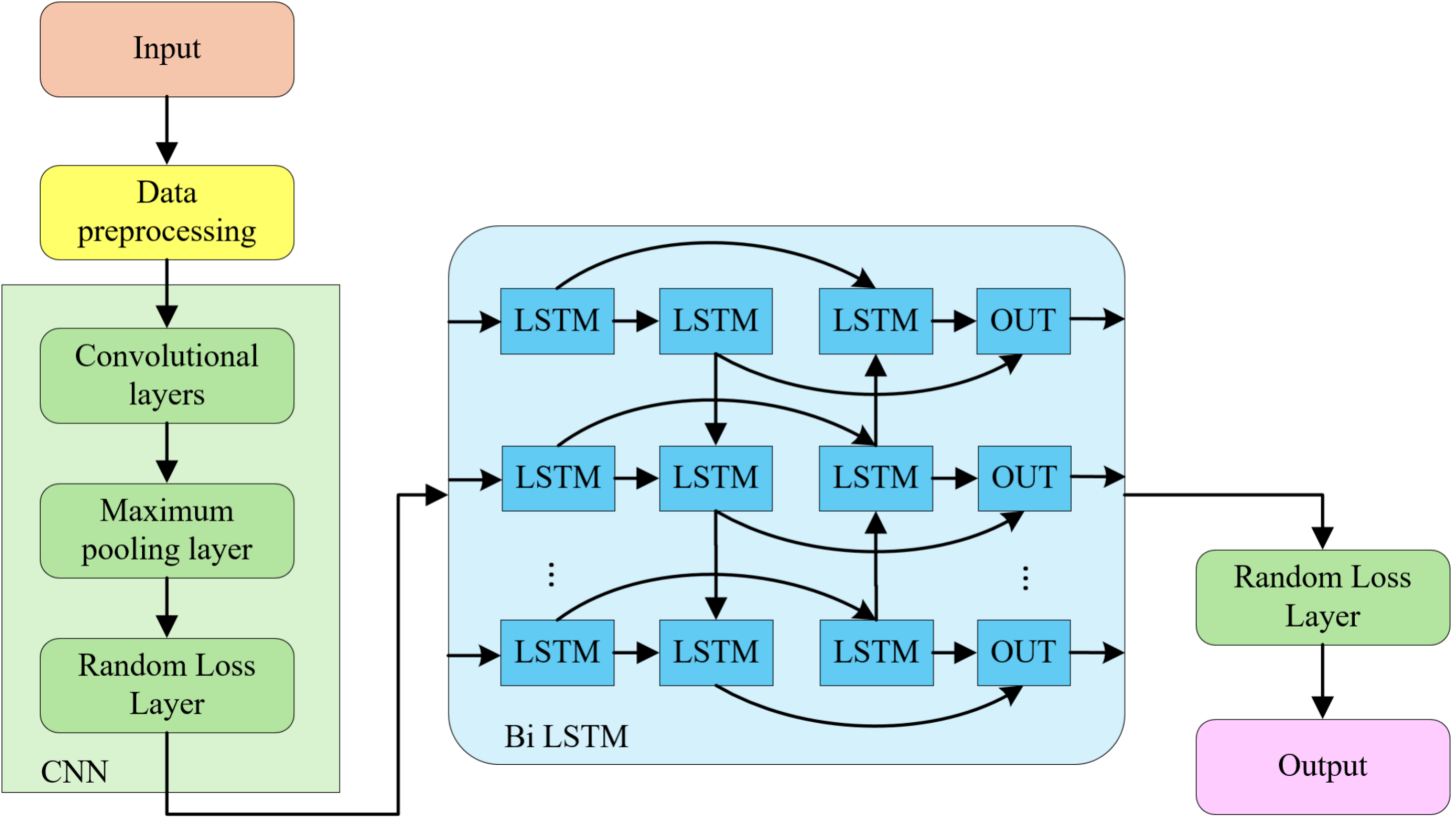
TS-CNN-BiLSTM model structure.

TS-CNN-BiLSTM is a deep learning model that incorporates a CNN and a bi-directional long short-term memory network (as shown in Figure 1). In this model, CNN is responsible for extracting features from high-dimensional data, such as images or other types of data. BiLSTM is specialized in processing temporal features, such as text or time series data. Both models have strong nonlinear fitting capabilities and can automatically extract key features from the data. By fusing CNN and BiLSTM, the advantages of deep learning models in feature extraction can be fully utilized.

The BiLSTM layer processes sequential data bidirectionally, capturing both long-term dependencies (e.g., weekly exercise trends) and short-term fluctuations (e.g., hourly activity bursts). Temporal inputs are segmented into sliding windows and encoded with cyclical features (e.g., sine/cosine transformations for time-of-day) to model periodic behaviors. Combined with CNN’s local feature extraction, this hybrid architecture ensures robust modeling of temporal dynamics, as evidenced by the model’s ability to identify critical patterns.

The convolutional layer is a fundamental component of the CNN, which is responsible for extracting meaningful features from input data such as images, text, or other high-dimensional data. The convolutional kernel, represented by the matrix $m_{w,t}$ , is a trainable parameter that defines the receptive field of the convolutional layer. This kernel slides over the input data, performing element-wise multiplications and summations to extract localized features.

Role of the Convolutional Kernel: (1) Feature Extraction: The convolutional kernel acts as a filter that captures specific patterns or features in the input data. For example, in image processing, different kernels can detect edges, textures, or other visual patterns. In the context of the TS-CNN-BiLSTM model, the convolutional kernel helps in identifying temporal and spatial patterns in the life log data. (2) Receptive Field: The size of the convolutional kernel (e.g., 3×3, 5×5) determines the receptive field, which is the region of the input data that each neuron in the convolutional layer is connected to. A smaller kernel size captures fine-grained details, while a larger kernel size captures broader patterns. (3) Nonlinear Activation: After the convolutional operation, the result is passed through a nonlinear activation function (e.g., ReLU) to introduce nonlinearity into the model. This allows the network to learn more complex patterns and representations.

The formula for the convolutional operation is as follows in Equation 1:


(1) $$g_{x,y}=f\left(\overset{W}{\underset{w=1}{\sum }}\;\overset{T}{\underset{t=1}{\sum }}\;m_{w,t}\cdot i_{x+w,y+t}+h\right)$$

Where $g_{x,y}$ is the corresponding activation, $m_{w,t}$ is the w×t matrix of the convolution kernel, and $i_{x+w,y+t}$ represents the input at neuron (*x*,*y*). *h* is the bias value and *f* is the nonlinear function. In this paper, the convolutional layer uses a rectified linear unit (ReLU) to compute the feature mapping, and its nonlinear function is defined as shown in Equation 2:


(2) $$\sigma \left(i\right)=\max\left(0,i\right)$$

ReLU is chosen for its computational efficiency and ability to mitigate gradient vanishing in deep networks. Unlike saturating activation functions (e.g., sigmoid), ReLU maintains a non-zero gradient for positive inputs, accelerating convergence during training. Additionally, its sparsity-inducing property (zero output for negative values) reduces overfitting and enhances model robustness. These characteristics make ReLU particularly suitable for extracting discriminative features from multimodal life log data while ensuring efficient training.

Pooling layer: the feature vectors output from the convolutional layer usually have high dimensions, which will cause greater computational pressure on the model. The pooling layer is mainly responsible for selecting and filtering the feature data extracted from the convolutional layer, which can reduce the dimensionality of the information obtained by the convolutional layer. It can not only alleviate the computational pressure on the model, but also prevent the model from over fitting. There are three common pooling operations, max pooling, minimum pooling and average pooling, and the model in this paper uses maximum pooling. The principle of max pooling is to select the largest feature value in each sub-region of the input data as the output. Specifically, max pooling splits the input data into non-overlapping regions (usually rectangular regions) and then selects the largest feature value in each region. This reduces the dimensionality of the data and retains the most salient features. The formula for maximum pooling can be expressed as follows in Equation 3:


(3) $$\max\mathrm{Pooling}\left(I_{x,y}\right)=\max\left(I_{x,y}\left[u,v\right]\right)$$

Where $I_{x,y}$ is the sub-region of the *x*-th row and *y*-th column of the input data, and $I_{x,y}\left[u,v\right]$ denotes the elements of the *u*-th row and *v*-th column in the sub-region.

In the BiLSTM model, the input sequence is split into two directions, i.e., forward and reverse. And then an LSTM cell is constructed in each direction. This structure allows the model to take contextual information into account simultaneously, which improves the performance of the model. The output features of the CNN are spliced together to form a longer feature vector, which is then passed to the BiLSTM. The BiLSTM model structure can be represented by the following Equations 4–8:


(4) $$x_{n}=\sigma \left(W_{i_{x}}i_{n}+W_{b_{x}}b_{n-1}+h_{x}\right)$$

(5) $$f_{n}=\sigma \left(W_{i_{f}}i_{n}+W_{b_{f}}b_{n-1}+h_{f}\right)$$

(6) $$c_{n}=f⊙c_{n-1}⊙\tanh\left(W_{i_{c}}i_{n}+W_{b_{c}}b_{n-1}+h_{c}\right)$$

(7) $$o_{n}=\sigma \left(W_{i_{o}}i_{n}+W_{b_{o}}b_{n-1}+h_{o}\right)$$

(8) $$b_{n}=o_{n}⊙\tanh\left(c_{n}\right)$$

Where $x_{n}$ , $f_{n}$ , and $o_{n}$ are the outputs of the input, forget, and output gates, respectively. $c_{n}$ is the update of the cell state, and $b_{n}$ is the update of the hidden state. *Tanh* and *σ* are the activation functions, and ⊙ is the element-by-element multiplication operation. $i_{n}$ is the input of the current time step, and $b_{n-1}$ is the hidden state of the previous time step. $M_{ix}$ , $M_{i_{f}}$ , $M_{i_{c}}$ , $M_{i_{o}}$ and $M_{b_{x}}$ , $M_{b_{f}}$ , $M_{b_{c}}$ , $M_{b_{0}}$ are the weight matrices of the input/output and hidden states, respectively. $h_{x}$ , $h_{f}$ , $h_{c}$ , $h_{o}$ are the bias terms. In the TS-CNN-BiLSTM model, the output layer consists of a fully connected layer and a Soft Max classifier. Each node of the fully connected layer is connected to a node in the upper layer so that features extracted from the upper layer can be merged. Following the fully connected layer is the Soft Max classifier, which converts the output of the upper layer into a probability vector whose value represents the probability of the class to which the current sample belongs. In a multi-classification task, a cross-entropy loss function is used as the loss function of the model. If the labels are in the form of unique heat coding, Categorical Cross entropy is usually used. The categorical labels used in this paper are in the form of integers (non-unique heat coding), and Sparse Categorical Cross entropy is used as the loss function, which is calculated as follows in Equation 9:


(9) $$B\left(j,\hat{j}\right)=-\underset{x}{\sum }\;j_{x}\log\left(\hat{j}\right)$$

Where *j* is the integer form of the true label, $\hat{j}$ is the predicted probability distribution vector of the model. The summation $\underset{x}{\sum }$ iterates over all classes, and $j_{x}$ is a binary indicator (0 or 1) that equals 1 if the true class is x and 0 otherwise. In this equation, the loss function computes the negative log-likelihood of the true class given the predicted probabilities. This ensures that the model is penalized more for being confident about incorrect predictions and less for being uncertain about the correct predictions.

## 4 Results analysis and discussion

### 4.1 Model setup

In this study, the loss function used for network model training is cross entropy loss function, and the gradient descent optimization algorithm adopts Adam, in which the parameters of β1 and β2 are taken as 0.900 and 0.999. The small batch gradient descent method with a batch size of 64 can make full use of the computational resources of the machine and accelerate the convergence speed of network model training. A learning rate decay strategy is used, where the initial learning rate is set to 0.001 and the learning rate is halved every 10 iterations. This helps the convergence of network model training and makes it easier to obtain the optimal network model. The early stop strategy is to stop training when the loss function value of the validation set increases within 10 consecutive iterations during the training process.

The network model is trained and validated using 10-fold cross-validation, i.e., the entire dataset is equally divided into 10 pieces of data. A different 9 copies and the remaining 1 copy are selected as the training set and validation set, respectively, for each cross-validation training. The specific procedure of 10-fold cross-validation is as follows: firstly, the order of the dataset is disorganized; then the location index of each sample in the disorganized dataset is saved; finally, 8 times training and validation of the network model are carried out on the disorganized dataset where the sample indexes are saved, and 8 network models are obtained.

The performance metrics used in this study include accuracy, precision, recall, F1 score, and confusion matrix. Accuracy, precision, and recall are the performance of the network model in terms of “finding the right,” “finding the right,” and “finding the right,” respectively; the F1 score is the reconciled average of the precision and recall. The formulas for, precision accuracy on, recall and F1 score are as shown in Equations 10–13:


(10) $$\mathrm{Accuracy}=\frac{TP+TN}{TP+TN+FP+FN}$$

(11) $$\mathrm{Precision}=\frac{TP}{TP+FP}$$

(12) $$\mathrm{Recall}=\frac{TP}{TP+FN}$$

(13) $$F_{1}=\frac{2\times \mathrm{precision}\times \mathrm{recall}\;}{\;\mathrm{precision}+\mathrm{recall}\;}$$

**Figure 2:**
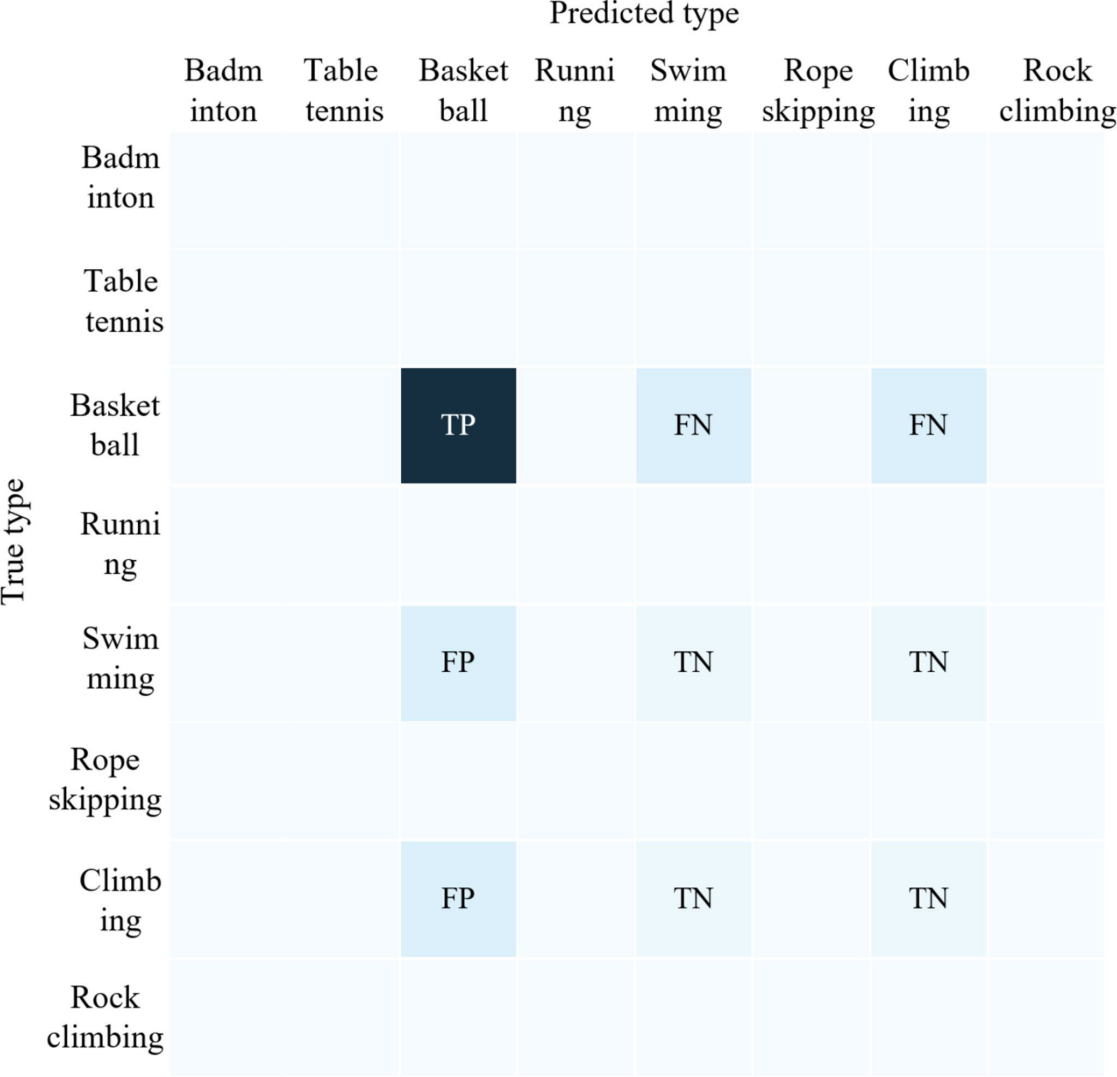
Schematic diagram of the samples included in TP, FP, TN, and FN when predicting the type of rope skipping.

True Positive (*TP*) is the number of samples that the model correctly predicted as belonging to a particular type of physical activity, True Negative (*TN*) is the number of samples that the model correctly identified as “not in this category” False Positive (*FP*) is the number of samples that the model incorrectly predicted as not belonging to a particular type of physical activity, False Negative (*FN*) is the number of samples that the model incorrectly predicted as belonging to other physical activity types. False Positive (*FP*) is the number of samples that the model incorrectly predicts as not belonging to a particular sport and exercise type, and False Negative (*FN*) is the number of samples that the model predicts as other sport and exercise types. Taking basketball as an example, the samples contained in the four variables *TP*, *TN*, *FP*, and *FN* are shown schematically in Figure 2.

The process of analyzing the network models using the above four performance metrics in this study was as follows: first, the eight network models trained were classified for each of the eight physical activity type samples in their validation set; then the above four performance metrics were averaged for the classification results; and finally, a comparison was made with the results of the current state-of-the-art research. When performance comparisons are made between network models, the network models are considered to perform better when the accuracy, precision, recall and F1 score all perform higher. The confusion matrix is a matrix with *N* rows and *N* columns. The columns represent the predicted types of physical activity and the rows represent the true types of physical activity. The cell values indicate the number of samples where the true type is the class represented in the row and the network model predicts it as the class represented in the column.

### 4.2 Analysis of results

#### 4.2.1 Classification of physical activity types

**Figure 3:**
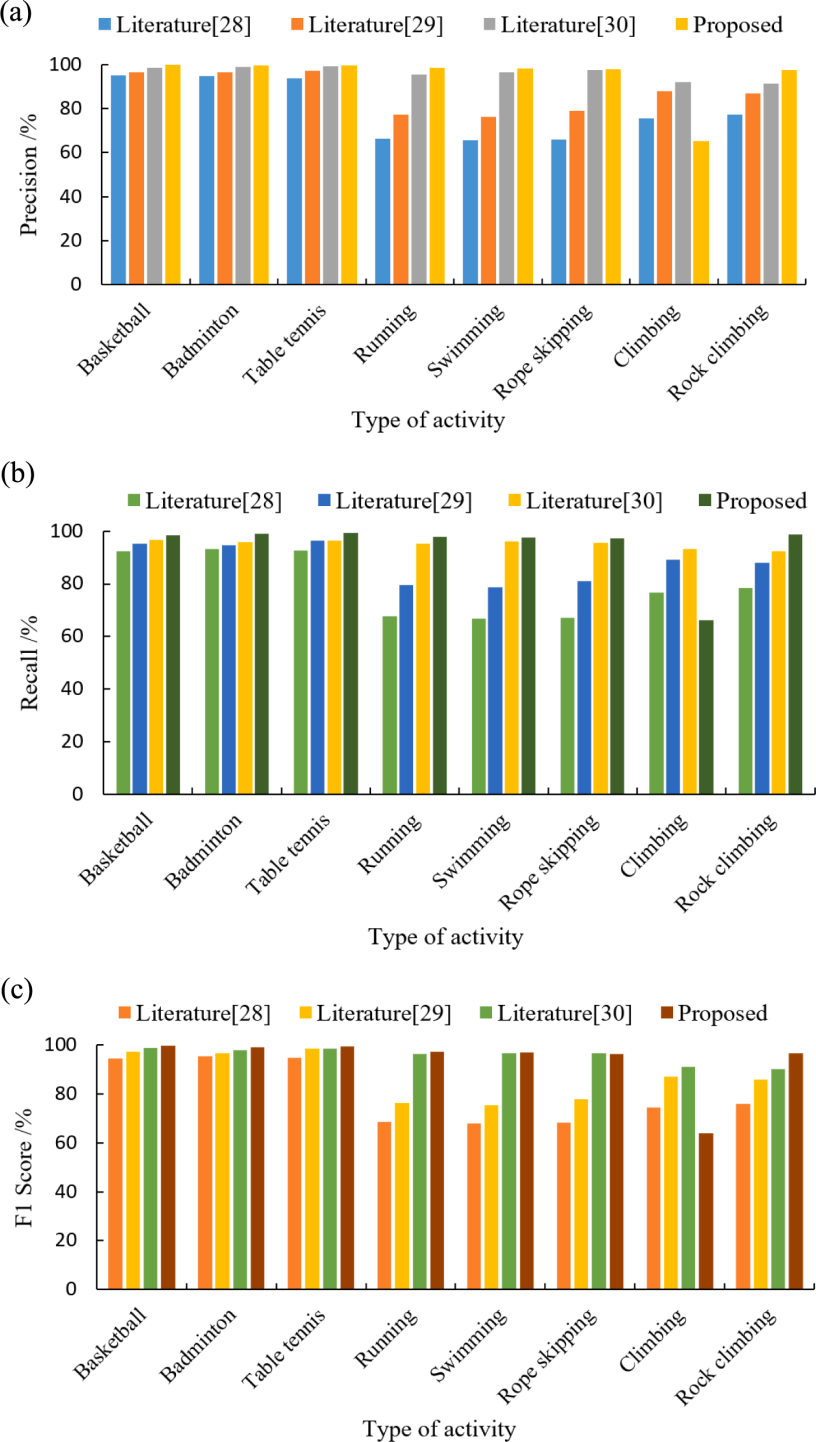
Performance evaluation of different models to classify eight physical exercise types. **(a)** Precision. **(b)** Recall. **(c)** F1 score.

**Figure 4:**
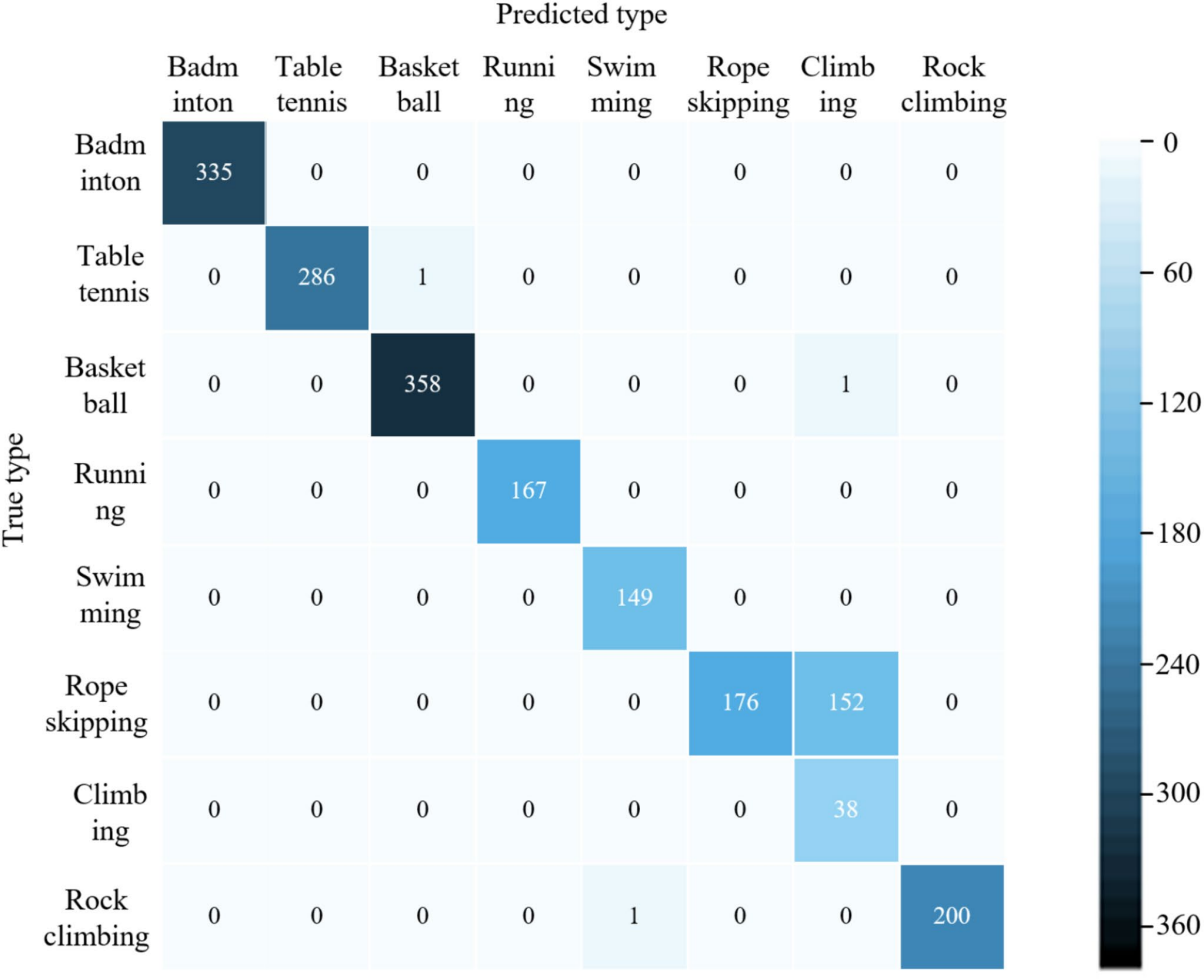
Figure confusion matrix for classifying samples in the validation set using the proposed model.

Figure 3 compares the precision, recall, and F1 scores of our hybrid model against literatures (28–30) for eight physical activity categories. As can be seen in Figure 3, the performance metrics of the four models for categorizing ball sports activities (basketball, badminton, and table tennis) are higher than those of other physical activity types. The mean values of the three metrics of the proposed model are (99.8, 98.9, and 99.5%). The models trained in literature (28), literature (29) and literature (30) were (94.7, 92.7, 94.9%), (96.9, 95.4, 97.6%) and (98.8, 96.2, 98.4%) respectively. The model classification performance of this paper outperformed the other 3 methods. In terms of aerobic exercise (running, swimming, and jumping rope) classification, the model performance of literature (28) training was generally low, with the mean values of the 3 metrics being (66.1, 67.2, and 68.3%). Literature (29) model improved compared to literature (28) with 77.6, 79.8, and 76.5%, respectively. The mean values of the three metrics for the literature (30) model and the proposed model are (96.5, 95.7, 96.7%) and (98.2, 97.6, 96.9%), respectively. The classification averages of both models are higher than 95%, while the proposed model performs better. For the classification of outdoor physical activities, the mean values of the three metrics of the model of literature (30) were (91.8, 92.9, 90.7%), which had the highest overall performance and the best classification performance. Literature (29) and the proposed model were the next highest, and literature (28) model had the lowest performance. As the acceleration of hill climbing and rope skipping were closer in the collected data, the two activities were also very close in terms of amplitude and repetition period. From the confusion matrix, it can be seen that the proposed model predicts a large amount of hill climbing as rope skipping (as shown in Figure 4), and thus has a low classification performance for the climbing type. Overall, the proposed model has good classification performance for all other physical activity types.

#### 4.2.2 Classification by physical activity type combined

**Figure 5:**
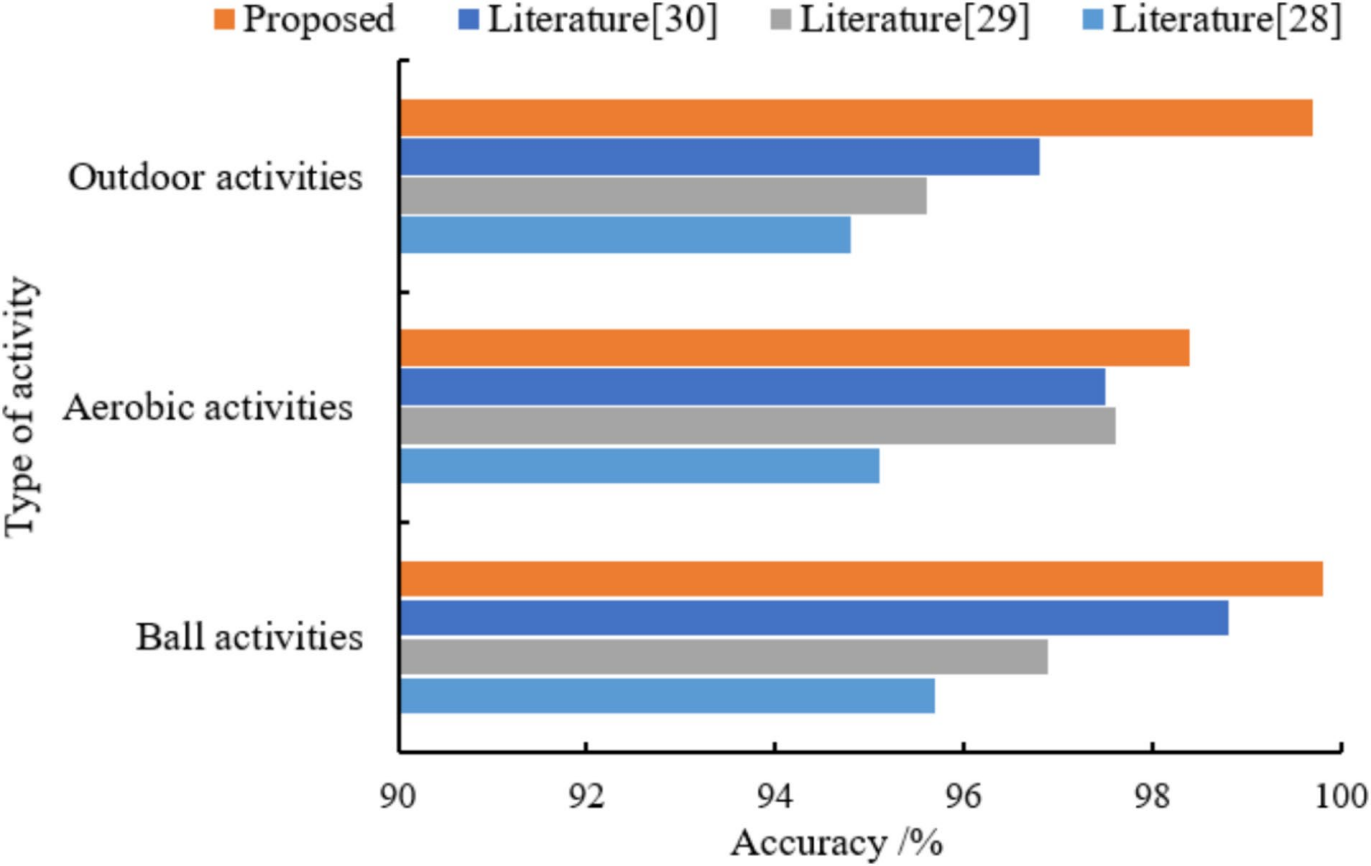
Comparative results of the accuracy of different models.

The 8 types of physical activity were classified into three major categories according to different types: ball sports, aerobic sports and outdoor sports. The training convergence of the models classified under the three types of physical activity was faster than that of the eight types of physical activity. The results of the comparison of the three metrics of the different models under the three classifications are shown in Table 3, and the results of the accuracy comparison are shown in Figure 5.

**Table 3:** Performance evaluation of different network models for categorization of three physical activity types.

Metrics	Types	Literature (28)	Literature (29)	Literature (30)	Proposed
Precision	Ball activities	94.7	96.9	98.8	99.8
	Aerobic activities	66.1	77.6	96.5	98.2
	Outdoor activities	76.4	87.6	91.8	81.5
Recall	Ball activities	92.7	95.4	96.2	98.9
	Aerobic activities	67.2	79.8	95.7	97.6
	Outdoor activities	77.5	88.7	92.9	82.6
F1 score	Ball activities	94.9	97.6	98.4	99.5
	Aerobic activities	68.3	76.5	96.7	96.9
	Outdoor activities	75.3	86.5	90.7	80.4

**Figure 6:**
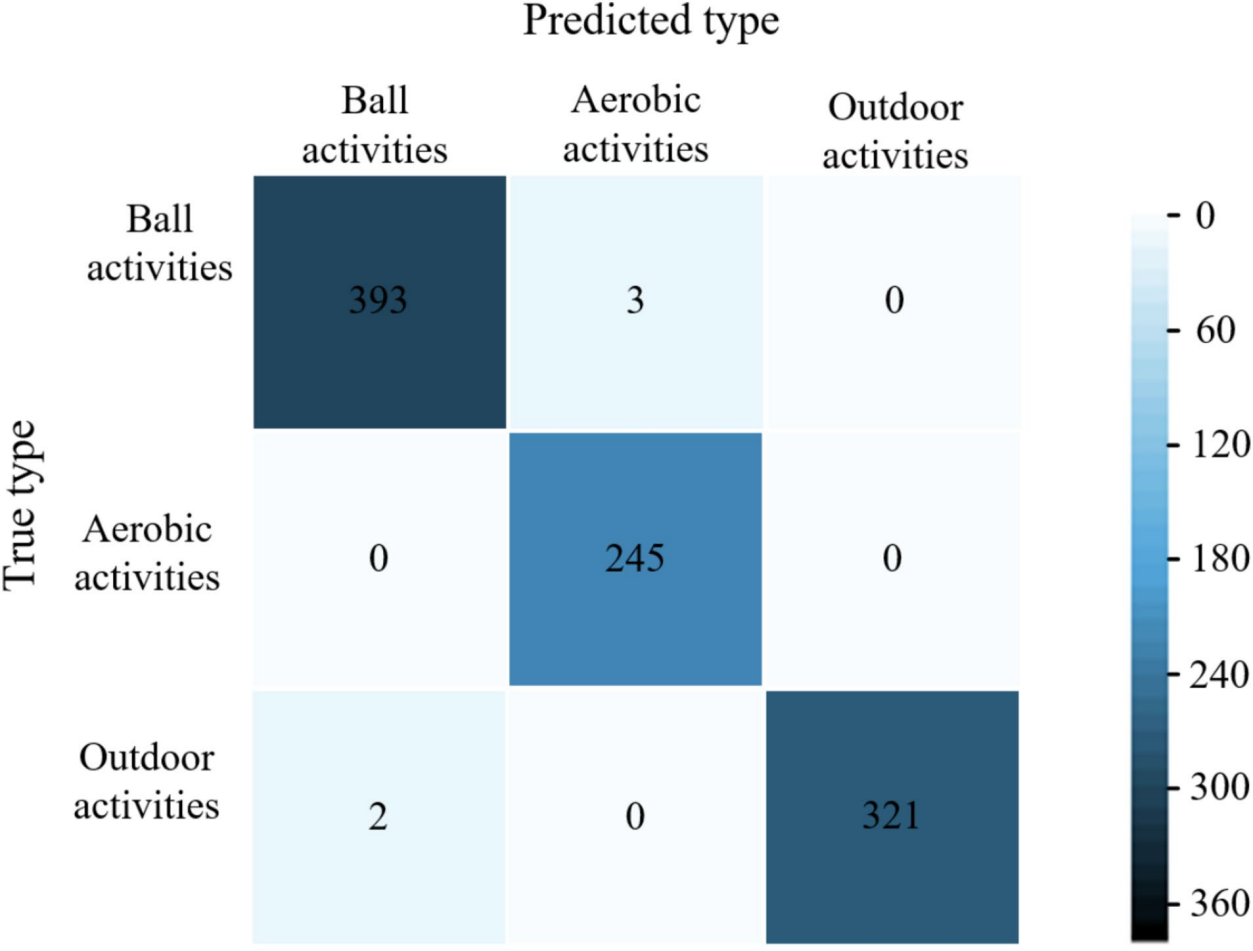
Confusion matrix of the proposed model for classification of validation set samples.

**Figure 7:**
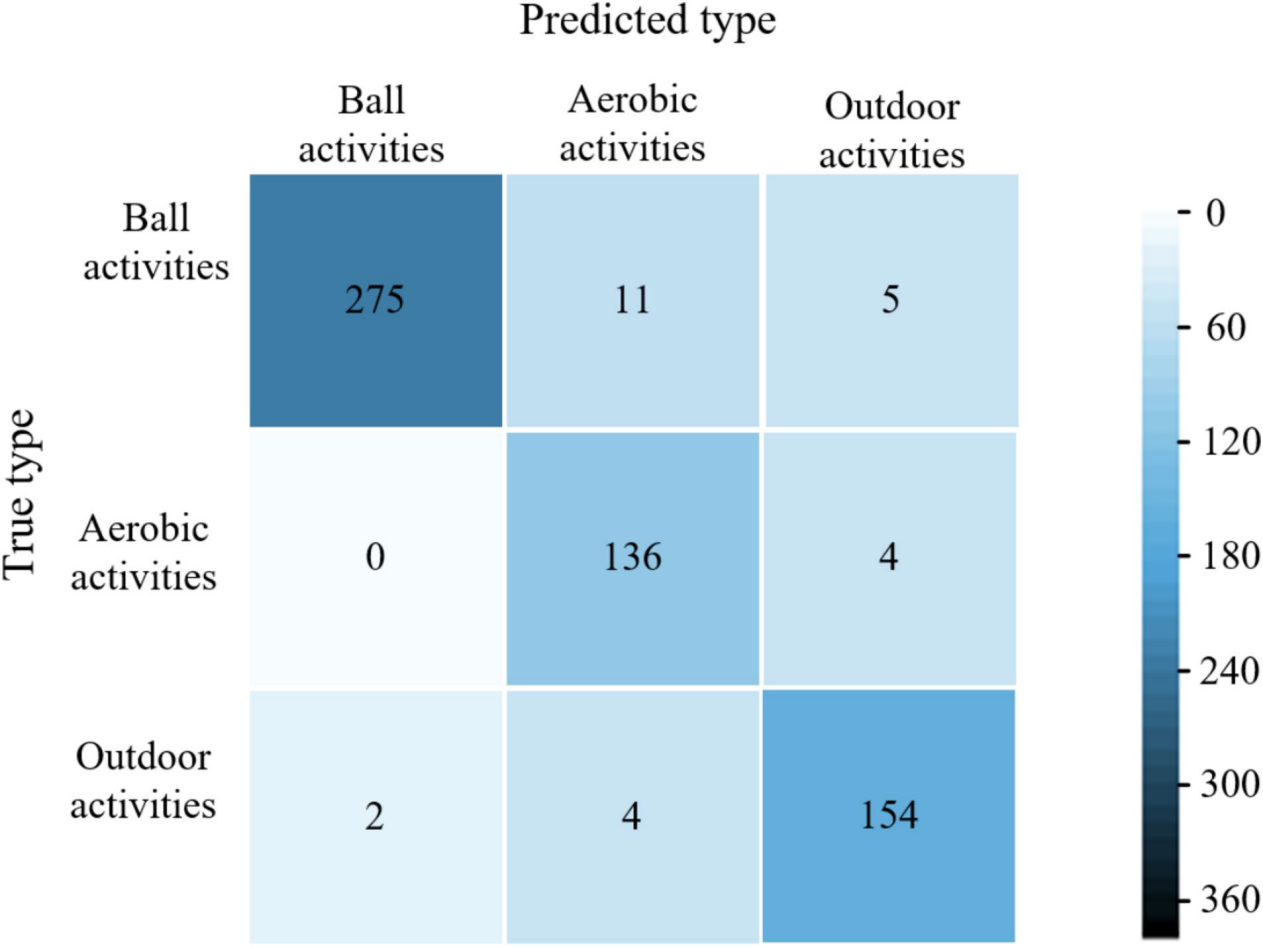
Confusion matrix for classification of validation set samples by the model of literature (28).

**Figure 8:**
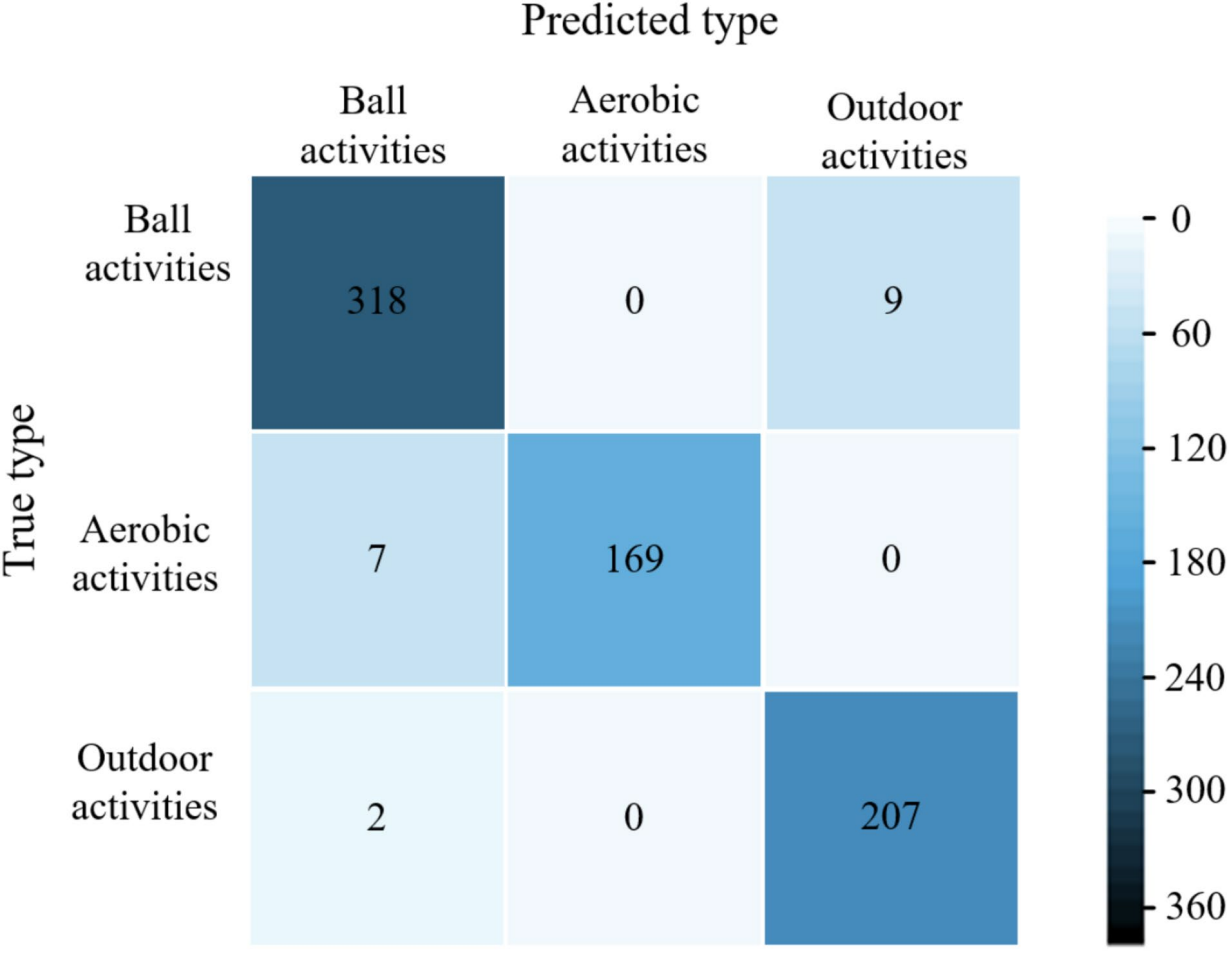
Confusion matrix of the model in literature (29) for sample classification in the validation set.

**Figure 9:**
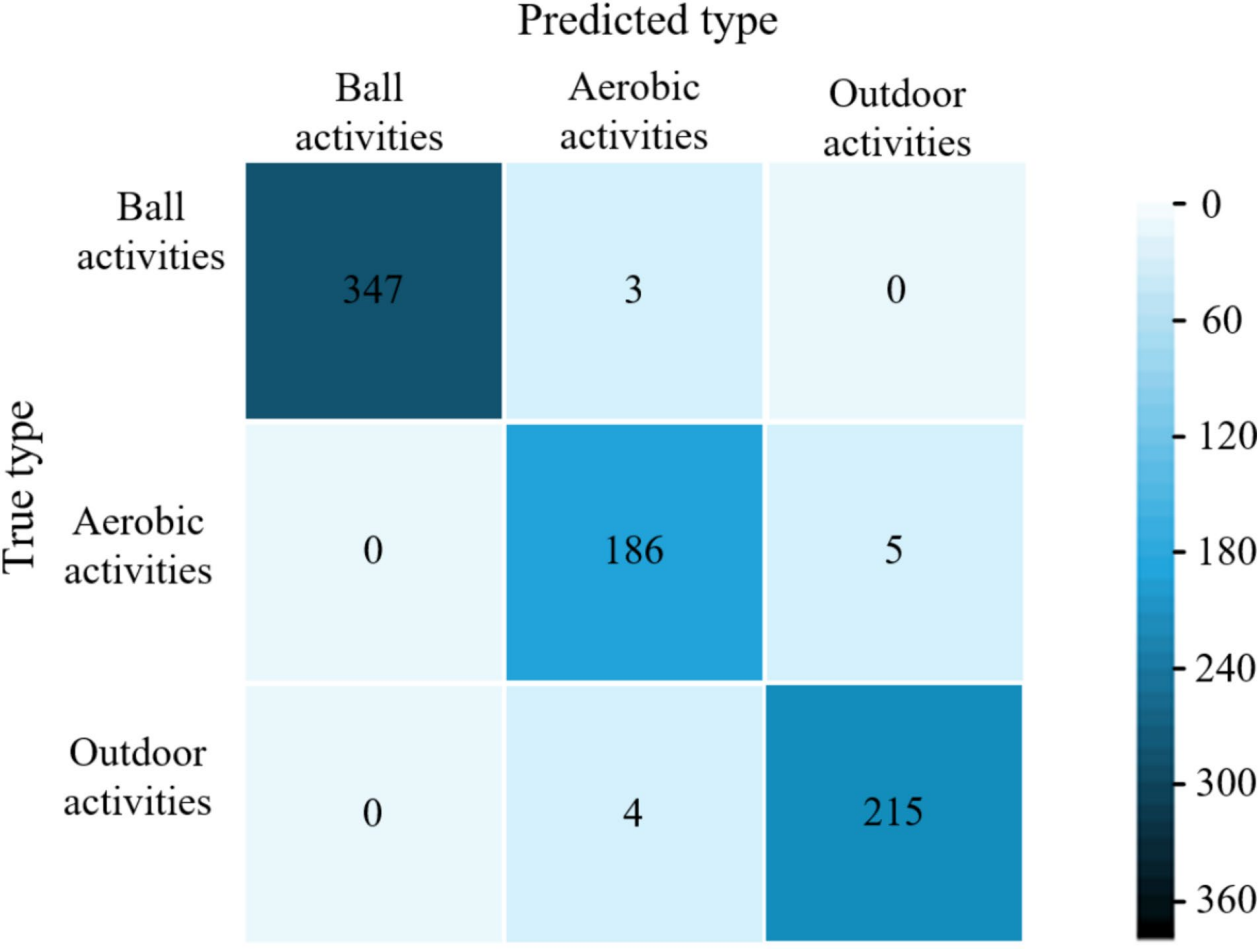
Confusion matrix for classification of validation set samples by the model of literature (30).

The average accuracy of the proposed model is 99.6%, which is 4.4, 2.9, and 1.9% higher than the average accuracy of the models in literature (28), literature (29) and literature (30), respectively. The experimental results show that the proposed models have excellent classification performance for all three types of physical activity. The confusion matrices of different models for classifying validation set samples are shown in Figure 6–9. The optimal network model trained in this study misclassified only 5 of all samples in its validation set. In contrast, the optimal network models trained in literature (28), literature (29) and literature (30) classified all samples in its validation set with 26, 18, and 12 errors, respectively.

#### 4.2.3 Comparison of classification results on different datasets

**Figure 10:**
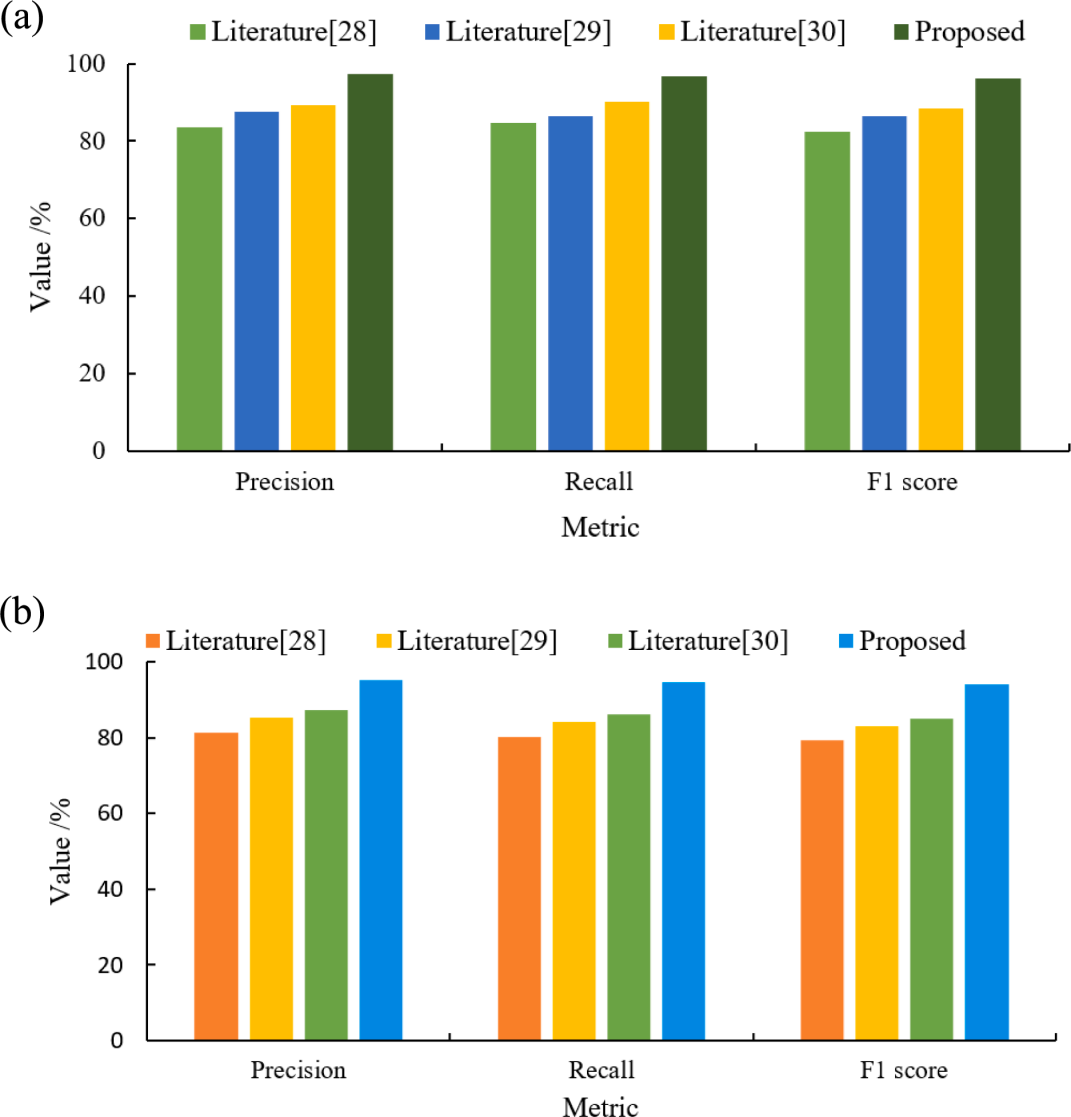
Classification results of the four models under different datasets. **(a)** Geo-Life Log. **(b)** TS-Life Log.

In order to deeply explore the role of user location information in behavioral classification, two datasets with different features were constructed in this study with the aim of assessing the specific impact of location information on the accuracy of user behavioral classification. Each of these two datasets integrates a different combination of features, one of which contains the user’s location information while the other does not, thus allowing us to analyze the extent of the contribution of location information in model performance against each other. The experimental results are shown in Figure 10.

Analyzing the data in Figure 10 shows that on the same Geo-Life Log or Time-Life Log dataset, the TS-CNN-BiLSTM model proposed in this paper has better classification results than the other three models. This is because the BiLSTM model in the hybrid model of this paper can utilize the bidirectional contextual information to better capture text features. Meanwhile, the combination of CNN and BiLSTM in this study can better utilize the local and global information of the text to achieve better classification results. The performance of the models on the Time-Life Log dataset is generally slightly lower than that on the Geo-Life Log dataset. This is due to the fact that the Time-Life Log dataset has less textual feature information and the lack of location information causes the model to face greater challenges in the classification task. Despite the lack of location information in the Time-Life Log dataset, the model combining CNN and BiLSTM in this study is still able to effectively improve the classification performance in this case.

## 5 Health promotion strategies

In order to effectively promote the improvement of adolescents’ physical fitness and exercise behaviors, this study proposes a health promotion strategy based on the LLSM, which focuses on personalized interventions, behavioral incentives, and social support in order to achieve a sustained impact on the adolescent population. Specifically:

(1) Personalized intervention: The LLSM enables real-time tracking and analysis of adolescents’ physical activity data, including exercise frequency, duration, and type. Based on these data, personalized exercise advice and goal setting are provided to each adolescent to help him/her choose the right type of exercise according to his/her personal health status and interest preferences. In addition, combined with biofeedback technology, such as heart rate monitoring and other data, the exercise intensity and frequency can be finely adjusted to maximize the effect of exercise and avoid over-fatigue or sports injuries.
(2) Behavioral Incentive Mechanism: The LLSM motivates youth to maintain regular physical activity habits by setting up incentive mechanisms. For example, the model can issue points based on exercise performance, which can be exchanged for virtual rewards or actual prizes. Trends in exercise progress are identified through data analysis, and timely feedback on progress and achievements enhances adolescents’ motivation to exercise. In addition, the model can help teens stay motivated and self-confident during exercise by setting milestones.
(3) Social Support and Interaction: Social interaction has an important impact on the formation of behavioral habits of adolescents during their growth process. The LLSM enables adolescents to share exercise logs and health data with peers, parents or fitness trainers through the social platform function, thus establishing a supportive social network. In this social interaction, adolescents can encourage each other, share exercise experiences, and increase engagement and competitiveness through group challenges and team sports. The reinforcement of social support can help increase adolescents’ exercise adherence and reduce the risk of quitting exercise due to isolation or lack of external motivation.
(4) Synergistic interventions at home and school: The LLSM not only supports monitoring and intervention of individual adolescent behavior, but also provides a convenient health management tool for families and schools. Parents and teachers can use the platform to view trends in adolescent exercise behavior and make timely communication and adjustments. For example, parents can rationalize the time and content of family exercise based on their children’s exercise data to enhance the family exercise atmosphere, while schools can combine the exercise log data to develop physical education courses and activities that better meet students’ interests and needs. The dual support of families and schools can more comprehensively promote the formation of healthy exercise habits among young people.

## 6 Conclusion

In this study, we propose an innovative solution for improving adolescent physical fitness behaviors by combining the LLSM with deep learning techniques. By designing a TS-CNN-BiLSTM combined model, we achieve efficient classification and prediction of multimodal life log data, and verify the excellent performance of the model in experiments. The model effectively integrates the local and global feature information, which can guarantee the privacy security and improve the classification accuracy even when the user’s location information is not included. The results show that the introduction of the time factor is crucial for capturing the dynamic trends of youth exercise behavior. Compared with other state-of-the-art methods, the proposed model shows significant advantages in several performance metrics, especially in the classification of different types of physical activities. This study provides technical support for the improvement of adolescent physical health behaviors and develops sustainable health promotion strategies.

Despite the significant contributions of this study, there are several limitations that should be acknowledged. Firstly, the generalizability of our model may be limited by the specific characteristics of our dataset. Future research should test the model on diverse datasets to ensure its applicability across different populations and settings. Secondly, the interpretability of the model remains a challenge. Deep learning models are often considered “black boxes” making it difficult to understand how specific features influence predictions. Future work will focus on improving the interpretability of the model decision process. We will explore techniques such as attention visualization and feature contribution analysis to elucidate how specific data inputs, such as temporal patterns or activity descriptions, drive predictions. This work aims to bridge the “black box” gap, ensure transparency, and promote trust between end users and healthcare stakeholders. Moreover, future work will expand beyond the Liu-Life Log dataset to evaluate the model’s generalizability on external benchmarks (e.g., PAMAP2, USC-HAD) and explore its adaptability to diverse populations and sensor configurations.

## Data Availability

The original contributions presented in the study are included in the article/supplementary material, further inquiries can be directed to the corresponding authors.

## References

[1] Dane A Bhatia K. The social media diet: a scoping review to investigate the association between social media, body image and eating disorders amongst young people. PLoS Glob Public Health. (2023) 3:e0001091–1. doi: 10.1371/journal.pgph.0001091, PMID: 36962983 PMC10032524

[2] Kua Z Hamzah F Tan PT Ong LJ Tan B Huang Z. Physical activity levels and mental health burden of healthcare workers during COVID-19 lockdown. Stress Health. (2022) 38:171–9. doi: 10.1002/smi.3078, PMID: 34231968 PMC8420337

[3] Rosenkranz RR Ridley K Guagliano JM Rosenkranz SK. Physical activity capability, opportunity, motivation and behavior in youth settings: theoretical framework to guide physical activity leader interventions. Int Rev Sport Exerc Psychol. (2023) 16:529–53. doi: 10.1080/1750984X.2021.1904434

[4] Lim MS Molenaar A Brennan L Reid M McCaffrey T. Young adults’ use of different social media platforms for health information: insights from web-based conversations. J Med Internet Res. (2022) 24:e23656–6. doi: 10.2196/23656, PMID: 35040796 PMC8808344

[5] Chelladurai U Pandian S. A novel blockchain based electronic health record automation system for healthcare. J Ambient Intell Humaniz Comput. (2022) 13:693–703. doi: 10.1007/s12652-021-03163-3

[6] Rusmitaningsih FN Subarjah H Yustiana YR Saputra DR. Enhancing students’ engagement and motivation in physical education: the role of fitness tracker apps. Edu Sportivo. (2024) 5:274–88. doi: 10.25299/esijope.2024.vol5(3).17215, PMID: 40098707

[7] Shao J Wu D. Evaluation on algorithms and models for multi-modal information fusion and evaluation in new media art and film and television cultural creation. J Comput Methods Sci Eng. (2024) 24:3173–89. doi: 10.3233/JCM-247565

[8] Pakhare JD Uplane MD. Hybrid mayfly Lévy flight distribution optimization algorithm-tuned deep convolutional neural network for indoor–outdoor image classification. Int J Image Graph. (2024) 24:2450024–4. doi: 10.1142/S0219467824500244

[9] Huang J Ren L Zhou X Yan K. An improved neural network based on SENet for sleep stage classification. IEEE J Biomed Health Inform. (2022) 26:4948–56. doi: 10.1109/JBHI.2022.3157262, PMID: 35259120

[10] Chen Y Luktarhan N Lv D. LogLS: research on system log anomaly detection method based on dual LSTM. Symmetry. (2022) 14:454–74. doi: 10.3390/sym14030454, PMID: 40053772

[11] Ali A Khan A Ahmed M Jeon G. BCALS: Blockchain-based secure log management system for cloud computing. Trans Emerg Telecommun Technol. (2022) 33:e4272–2. doi: 10.1002/ett.4272

[12] Singh P Saman Azari M Vitale F Flammini F Mazzocca N Caporuscio M. Using log analytics and process mining to enable self-healing in the internet of things. Environ Syst Decis. (2022) 42:234–50. doi: 10.1007/s10669-022-09859-x

[13] Yeruva AR Ramu VB. AIOps research innovations, performance impact and challenges faced. Int J Syst Syst Eng. (2023) 13:229–47. doi: 10.1504/IJSSE.2023.133013

[14] Notaro P Haeri S Cardoso J Gerndt M. Logrule: efficient structured log mining for root cause analysis. IEEE Trans Netw Serv Manag. (2023) 20:4231–43. doi: 10.1109/TNSM.2023.3282270, PMID: 39573497

[15] Li X Chen P Jing L He Z Yu G. Swisslog: robust anomaly detection and localization for interleaved unstructured logs. IEEE Trans Depend Sec Comput. (2022) 20:2762–80. doi: 10.1109/TDSC.2022.3162857

[16] Gu S Rong G Zhang H Shen H. Logging practices in software engineering: a systematic mapping study. IEEE Trans Softw Eng. (2022) 49:902–23. doi: 10.1109/TSE.2022.3166924, PMID: 39573497

[17] Ribeiro R Trifan A Neves AJ. Lifelog retrieval from daily digital data: narrative review. JMIR Mhealth Uhealth. (2022) 10:e30517–7. doi: 10.2196/30517, PMID: 35499858 PMC9112086

[18] Tran LD Nguyen MD Nguyen BT Zhou L. Myscéal: a deeper analysis of an interactive lifelog search engine. Multimed Tools Appl. (2023) 82:37789–806. doi: 10.1007/s11042-023-15078-6

[19] Diraco G Rescio G Siciliano P Leone A. Review on human action recognition in smart living: sensing technology, multimodality, real-time processing, interoperability, and resource-constrained processing. Sensors. (2023) 23:5281–1. doi: 10.3390/s23115281, PMID: 37300008 PMC10255964

[20] Chen Y Xu L Cheng Z Zhang D Yang J Yin C. Progression from different blood glucose states to cardiovascular diseases: a prospective study based on multi-state model. Eur J Prev Cardiol. (2023) 30:1482–91. doi: 10.1093/eurjpc/zwad196, PMID: 37315161

[21] Zhong J Zhang P Dong Y Xu Y Huang H Ye R. Well-being and cardiovascular health: insights from the UK biobank study. J Am Heart Assoc. (2024) 13:e035225–5. doi: 10.1161/JAHA.124.035225, PMID: 39291465 PMC11681495

[22] Beccaluva EA Catania F Arosio F Garzotto F. Predicting developmental language disorders using artificial intelligence and a speech data analysis tool. Hum Comput Interact. (2024) 39:8–42. doi: 10.1080/07370024.2023.2242837

[23] Chaturvedi V Kaur AB Varshney V Garg A Chhabra GS Kumar M. Music mood and human emotion recognition based on physiological signals: a systematic review. Multimedia Systems. (2022) 28:21–44. doi: 10.1007/s00530-021-00786-6

[24] Echtioui A Zouch W Ghorbel M Mhiri C Hamam H. Classification of BCI multiclass motor imagery task based on artificial neural network. Clin EEG Neurosci. (2024) 55:455–64. doi: 10.1177/15500594221148285, PMID: 36604821

[25] Ben-Dor Cohen M Nahum M Traub Bar-Ilan R Eldar E Maeir A. Coping with emotional dysregulation among young adults with ADHD: a mixed-method study of self-awareness and strategies in daily life. Neuropsychol Rehabil. (2024) 34:1161–85. doi: 10.1080/09602011.2023.2279181, PMID: 37971947

[26] Hoang-Xuan N Trang-Trung HP Tran MK Le TC Nguyen ER Ninh VT. First-flexible interactive retrieval system for visual lifelog exploration. Multimed Tools Appl. (2023) 82:37877–902. doi: 10.1007/s11042-023-16287-9, PMID: 40125411

[27] Klotzbaugh R Fawcett J. Gender minority Persons' perceptions of peer-led support groups: a Roy adaptation model interpretation. Adv Nurs Sci. (2023) 46:59–74. doi: 10.1097/ANS.0000000000000417, PMID: 35213876 PMC9399302

[28] Bozkurt F. A comparative study on classifying human activities using classical machine and deep learning methods. Arab J Sci Eng. (2022) 47:1507–21. doi: 10.1007/s13369-021-06008-5

[29] Yang P Yang C Lanfranchi V Ciravegna F. Activity graph based convolutional neural network for human activity recognition using acceleration and gyroscope data. IEEE Trans Industr Inform. (2022) 18:6619–30. doi: 10.1109/TII.2022.3142315

[30] Sarkar A Hossain SS Sarkar R. Human activity recognition from sensor data using spatial attention-aided CNN with genetic algorithm. Neural Comput Appl. (2023) 35:5165–91. doi: 10.1007/s00521-022-07911-0, PMID: 36311167 PMC9596348

